# Cascaded Fabry-Perot interferometer with thin film based on Vernier effect

**DOI:** 10.1038/s41598-025-90749-y

**Published:** 2025-02-21

**Authors:** Ling Chen, Qiang Wu

**Affiliations:** 1 School of Physics Science and Information Technology, Liaocheng University, Liaocheng, 252059 China; 2 Faculty of Engineering and Environment, Northumbria University, Newcastle upon Tyne, U.K.

**Keywords:** Optical physics, Physics, Optical physics, Nonlinear optics

## Abstract

The Vernier effect is often utilized to boost the sensing ability of optical fiber sensors. In this paper, theoretical model of cascaded Fabry-Perot interferometer (FPI) with thin film based on Vernier effect is established. The sensitivities of the envelope spectra, thin film cavity and mixed cavity of air-thin film are analyzed qualitatively. According to the theoretical analysis, although sensitivity from mixed cavity of air-thin film is amplified, the value is equal to the sensitivity of sing thin film cavity. Experimental verification is carried out by an example of thin film named polydimethylsiloxane (PDMS) polymer. Herein, a new FPI constructed by air cavity from a hollow-core fiber, PDMS cavity, and air-PDMS mixed cavity is proposed and demonstrated. In order to facilitate the generation of the Vernier effect, the length of the PDMS cavity is intentionally designed shorter than the air cavity, making the free spectral range of the air-PDMS cavity and air cavity is approximately equal. The temperature change makes the refractive index and thermal expansion of PDMS change, while gas pressure change results in elastic deformation of PDMS. The Vernier envelope wavelength shifts with the temperature and gas pressure change. The proposed FPI features high temperature and gas pressure sensitivities of 3.07 nm/℃, and 23.07 nm/MPa, and a high magnification factor of 17 when the lengths of HCF and PDMS are 82.5 and 3.7 μm, respectively. The experimental results show that the temperature and pressure sensitivities of the cascaded FPI’s envelope spectra are equal to the sensitivity of a single thin film microcavity, and the theoretical calculation is in good agreement with the experimental verification. The theoretical model is also applicable to thin film prepared by other polymer materials. Additionally, the proposed FPI has good stability, reversibility, and repeatability, which is a good choice in the field of optical fiber sensing.

## Introduction

Vernier effect will happen and a periodic envelope will appear only when two interferometers have similar optical path length (OPL)^1,2^. The Vernier effect is an effective technique to achieve the enhancement of the sensitivity in the field of optical fiber sensors^3^. High sensitivity can be obtained by tracking the shift of the envelope^4^. Recently, many fiber-optic interferometer configurations by cascading or parallel connection have been popularly developed to produce the Vernier effect such as Fabry–Perot interferometer (FPI)^5^, Mach–Zehnder interferometer (MZI)^6^, Michelson interferometer^7–9^, and Sagnac interferometer (SI) loop^10^. Furthermore, the performance of optical fiber sensors has been greatly improved by employing the Vernier effect in many aspects. For example, Lang et al.. designed an optical fiber sensor formed by cascaded liquid-air FPI cavities for temperature sensing, whose sensitivity can be magnified to 39.21 nm/℃ around 35℃. However, the disadvantage of this structure is the temperature detection range is extremely limited^11^. Lin et al.. developed a sensor composed of dual side-hole fiber MZI for gas pressure detection, which has high response to target measurand of ~-60 nm/MPa and a magnification factor (*M*) of 7 times is obtained^12^. Gu et al.. cascaded the two taper-based in-line MZI to produce the Vernier effect for magnetic field measurement, which has higher sensitivity of -5.148, and − 5.782 nm/mT than the single MZI with a M of 4.8 times^13^. Li et al.. experimentally demonstrated a biosensor with Vernier effect induced by the birefringence effect, whose sensitivity is ultra-high with value of 35,823.3 nm/RIU and detection limit is as low as 1 ng/mL^14^. Deng et al.. proposed a sensor based on the Vernier effect for strain sensing and a sensitivity with value of ~ 28.11 pm/µε is obtained. However, expensive femtosecond laser equipment is required to induce refractive index (RI)-modified area^15^. Wu et al.. made use of SI loop to engender the Vernier effect for hydrogen detection with sensitivity of -14.61 nm/%. Compared with interferometer without the Vernier effect, the sensitivity is improved by 1.85 times^16^. Compared with other types of fiber-optic interferometers, FPI attracted growing interest due to the superiority of compact size, flexible probe type, and outstanding sensing performance^17^.

Regarding the temperature and gas pressure sensitivity of FPI based on quartz glass, the value is extremely low due to low thermo-optic coefficient (TOC) and thermal expansion coefficient (TEC), and large Young’s modulus. Nowadays, the configuration of FPI has gradually evolved by combining with various polymer materials such as polyvinyl chloride, photoresist, optical adhesive, ultraviolet glue, and polydimethylsiloxane (PDMS)^18^. For example, sun et al.. designed a FPI based on ultraviolet curable liquid (NOA65) for simultaneous measurement of gas pressure and temperature with sensitivities of 1130 pm/MPa and 249 pm/°C, respectively^19^. However, the above FPI sensors with polymer materials have relatively low sensitivity. Wei et al.. presented a photoresist (IP-Dip, Nanoscribe) based 3D-printed miniature FPI. The fabricated device with a 140 μm cavity length has gas pressure and temperature sensitivities of 3.959 nm/MPa and 160.2 pm/°C^20^. Chen et al.. proposed a Two-photon 3D printing FPI sensor formed by the photoresist for simultaneous measurement of temperature and gas pressure by employing FBG, with gas pressure and temperature sensitivities of 6.6649 nm/MPa and 0.105 nm/°C, respectively^21^. However the equipment used in femtosecond laser-induced two-photon polymerization technology is very expensive.

Among them, PDMS, as a common polymer material, has the merit of high transparency, good light transmission, low cost, and good chemical stability. In addition, PDMS is a widely adopted temperature-sensitive material because of its high negative TOC of -4.66 × 10^− 4^/℃ and TEC of 9.6 × 10^− 4^/℃, which is often considered as the sensing FPI to enhance the temperature sensitivity^22^. Moreover, PDMS is an excellent candidate for the gas pressure detection. The high Poisson’s ratio and low elastic modulus make PDMS have excellent elastic deformation^23^.

In this study, a cascaded FPI with the Vernier effect by welding the single-mode fiber (SMF) and hollow core fiber (HCF) partially filled with PDMS is proposed and experimentally investigated for temperature and gas pressure sensing. By selecting a shorter PDMS cavity length, the optical path length of the air cavity is closer to that of the air-PDMS mixed cavity, thereby generating the Vernier effect and greatly improving the sensitivity. Temperature change increases the length of air-PDMS mixed cavity, decreases its RI, and also makes the length of air cavity reduce. The four samples are designed and fabricated for experimental research on temperature and gas pressure detection. Among them, the proposed FPI with 3.7 μm ultra-thin PDMS film and 82.5 μm HCF has the highest temperature and gas pressure responses with value of 3.07 nm/℃, and 23.07 nm/MPa, and the sensitivity is magnified by 17 times. A theoretical model with universal applicability to cascaded FPI with thin film based on Vernier effect is established. The theoretical calculation of sensitivity still applies when replaced with other thin film materials which will be instructive for the design of sensors in the future.

## Results

**Fig. 1:**
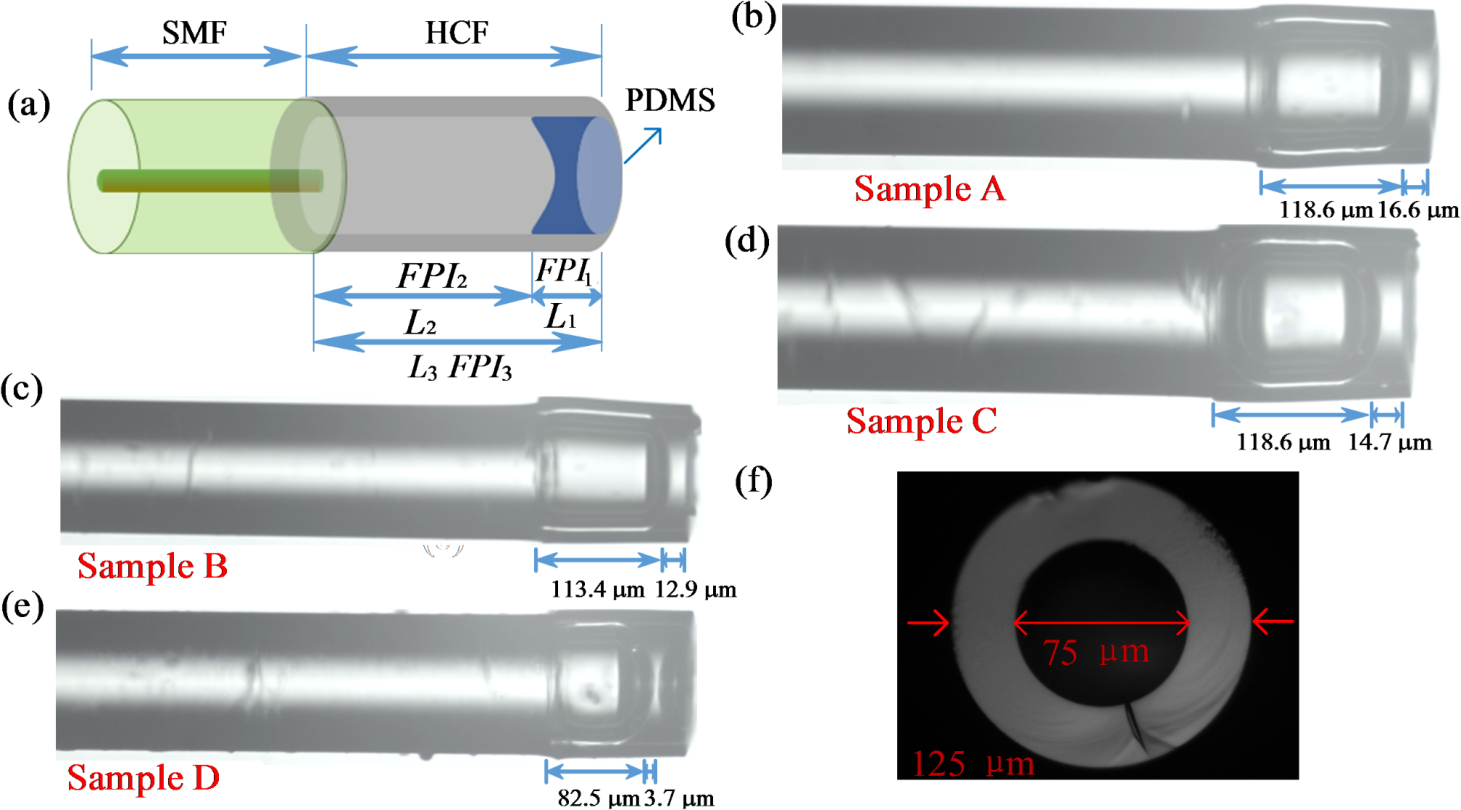
(**a**) Sensor structure of designed hybrid FPI; (**b**–**e**) Microscopic images of four hybrid FPIs (Samples A-D) with PDMS microcavity; (**e**) Microscopic images of HCF’s cross-section.

As presented in Fig. 1a, the proposed mixed FPI consists of three parts named SMF, HCF, and the PDMS microcavity, which includes air cavity (FPI_2_) with RI (n_2_ = 1) and length L_2_, PDMS microcavity (FPI_1_) with RI (n_1_) and length L_1_, and the mixed cavity (FPI_3_) with length L_3_. The length of the PDMS microcavity is smaller than that of the air cavity to make the OPLs of the air-PDMS hybrid cavity and air cavity as close as possible, resulting in the Vernier effect. The calculation method for the OPL is as follows: As shown in Fig. 1, the length of the air cavity and PDMS cavity can be calibrated using an optical microscope. The air cavity and PDMS microcavity lengths of samples A-D are 118.6, 113.4, 118.6, 82.5 μm, and 16.6, 12.9, 14.7, 3.7 μm, respectively. The calculation equation for the OPL is expressed as Eq. (1)^24^. Here, *n* and *L* are the refractive index and cavity lengths, respectively. Air and PDMS with refractive index values of 1.40 and 1 at room temperature^25^. Therefore, the OPLs of air cavity and PDMS cavity for four samples are calculated as 118.6, 113.4, 118.6, 82.5 μm, and 23.24, 18.06, 20.58, 5.18 μm, respectively. Figure 1b–e are the microscopic images of sample A-D, respectively. As presented in Fig. 1f, HCF has the inner and outer diameters of 75 and 125 μm.

1 $$OPL = nL$$

**Fig. 2:**
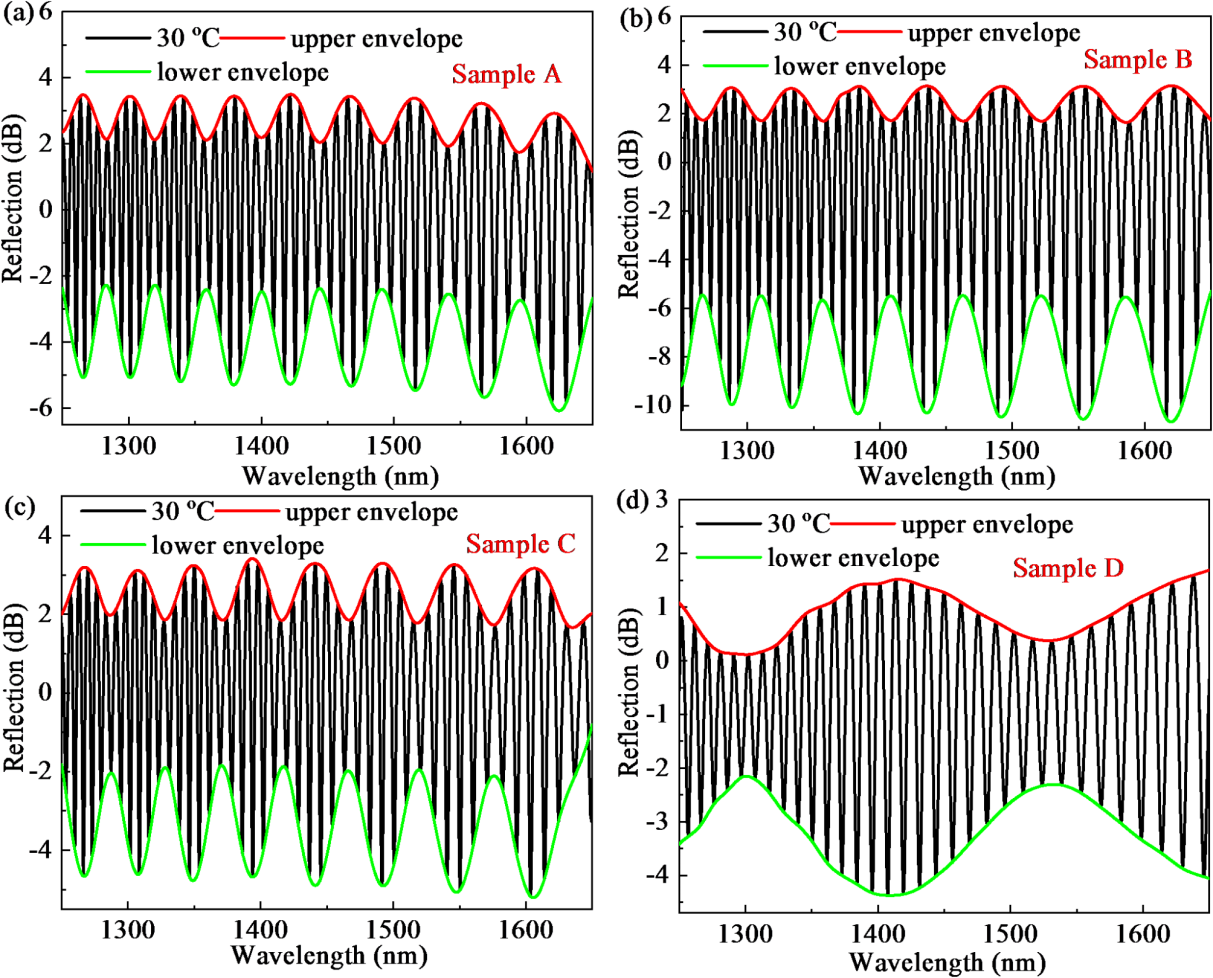
Reflection spectra of fabricated four cascaded FPIs with thin film based on Vernier effect at 30℃: (**a**) Sample A; (**b**) Sample B; (**c**) Sample C; (**d**) Sample D.

**Fig. 3:**
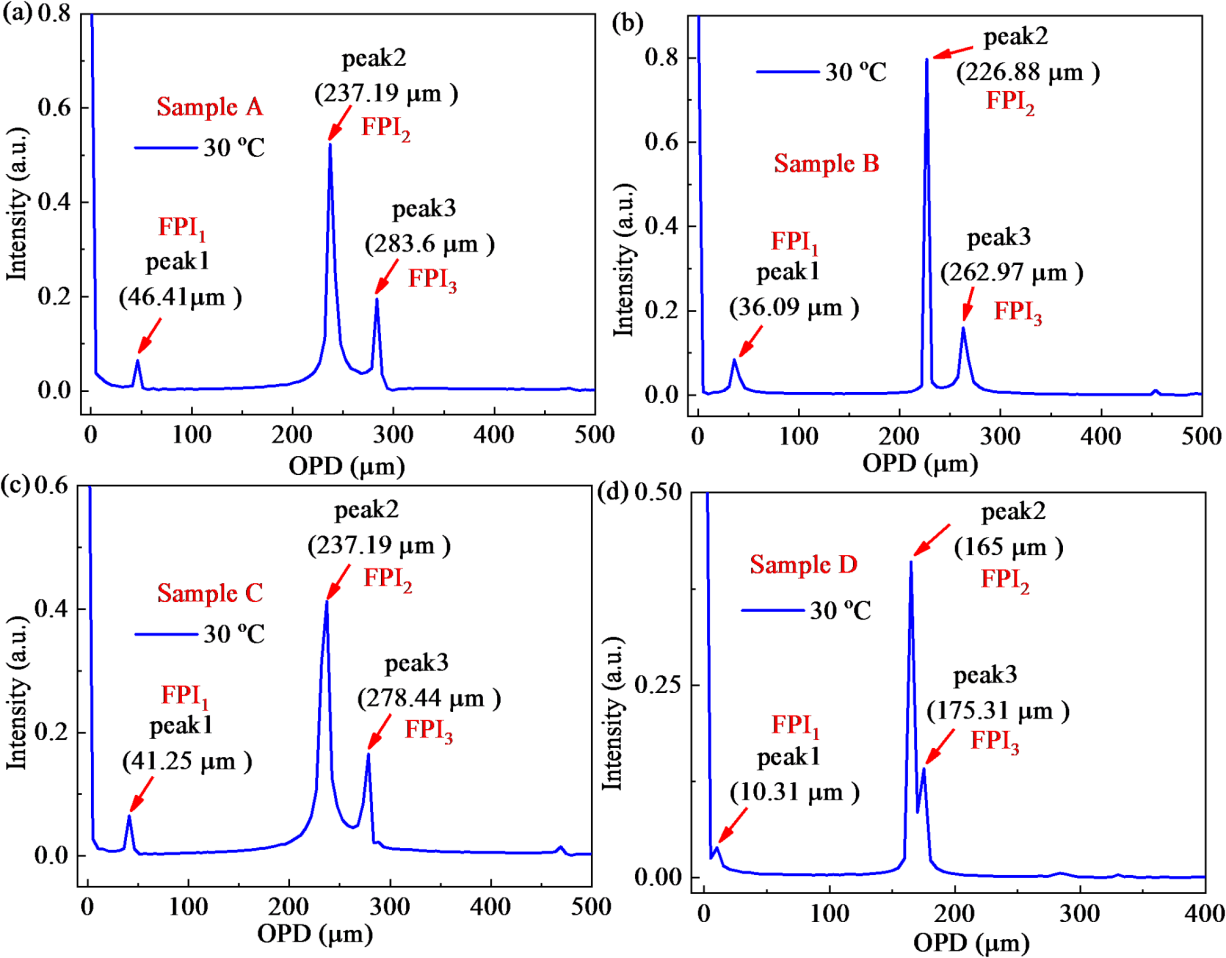
SFS based on FFT by the method of LWR using data in Fig. 2 as inputs: (**a**) Sample A; (**b**) Sample B; (**c**) Sample C; (**d**) Sample D.

The reflection spectra of fabricated four samples at 30℃ and the corresponding upper envelope and lower envelope are plotted in Fig. 2a–d, respectively. The Vernier effect is explicitly observed in Fig. 2a–d. To know exactly the interference component of designed FPI, the spatial frequency spectra (SFS) in Fig. 3a–d are obtained from Fig. 2a–d, respectively. A linear-in-wavenumber resampling (LWR) method based on fast Fourier transform (FFT) is employed^26^. From the SFS, we observed three peaks in the frequency domain, which means the air cavity from HCF (peak2, FPI_2_) with OPD_2_, PDMS microcavity (peak1, FPI_1_) with OPD_1_, and mixed cavity of the above two (peak3, FPI_3_) with OPD_3_, respectively. The value presented in Fig. 3a–d is twice the OPD and cavity length because of a one-way journal of the reflective structure. Therefore, the values of OPD_1_, OPD_2_, and OPD_3_ from samples A-D are 23.21, 118.6, 141.8; 18.05, 113.44, 131.49; 20.63, 118.6, 139.22 and 5.16, 82.5, 87.66 μm. The measured OPD is consistent with calculation results. As we all know, the traditional Vernier effect appears once the OPL of the two interferometers is closely matched. The superposition of spectra from the two paired interferometers will form a periodic envelope. The sensitivity can be magnified by tracking the spectral shift of the envelope. Here, the fundamental reason for the appearance of Vernier effect is due to the similar OPLs of the FPI_2_ and FPI_3_ of the four samples. The *M*, as a parameter for characterizing the performance of Vernier effect-based sensors, has closely link to the RI and cavity length of the air cavity and PDMS cavity expressed as Eq. (1)^27^:

2 $$M = \frac{{n_{1} L_{1} + n_{2} L_{2} }}{{n_{1} L_{1} }}$$

The *M* of samples A-D can be determined as 6.1, 7.3, 6.7, and 17 by incorporating specific parameters into formula (1). The FSR of the PDMS cavity, air cavity, and air-PDMS mixed cavity from four samples are 51.77, 10.13, 8.47 nm; 66.57, 10.59, 9.14 nm; 58.24, 10.13, 8.63 nm; and 233.03, 14.56, 13.7 nm by calculating by Eq. (2)^28^. Thus, the FSR of sample D is much larger than that of samples A–C. Table 1 lists the detailed parameters of the fabricated four samples.

3 $$FSR_{{PDMS}} \approx \frac{{\lambda _{m} ^{2} }}{{2n_{1} L_{1} }}{\text{ }}FSR_{{air}} \approx \frac{{\lambda _{m} ^{2} }}{{2n_{2} L_{2} }}{\text{ }}FSR_{{hybrid}} \approx \frac{{\lambda _{m} ^{2} }}{{2n_{1} L_{1} + 2n_{2} L_{2} }}$$

**Table 1:** Detailed parameters of four cascaded FPIs with thin film based on Vernier effect.

Sample	*OPD*_1_(µm)	*OPD*_2_(µm)	*OPD*_3_ (µm)	T. s. (dB/℃)	T. s. (nm/℃)	G. p. s. (nm/MPa)	M
A	23.21	118.6	141.8	0.026	1.32	4.74	6.1
B	18.05	113.44	131.49	0.049	1.51	4.87	7.3
C	20.63	118.6	139.22	0.022	1.48	4.77	6.7
D	5.16	82.5	87.66	0.018	3.07	23.07	17

### Theoretical sensitivity calculation of sensor

**Fig. 4:**
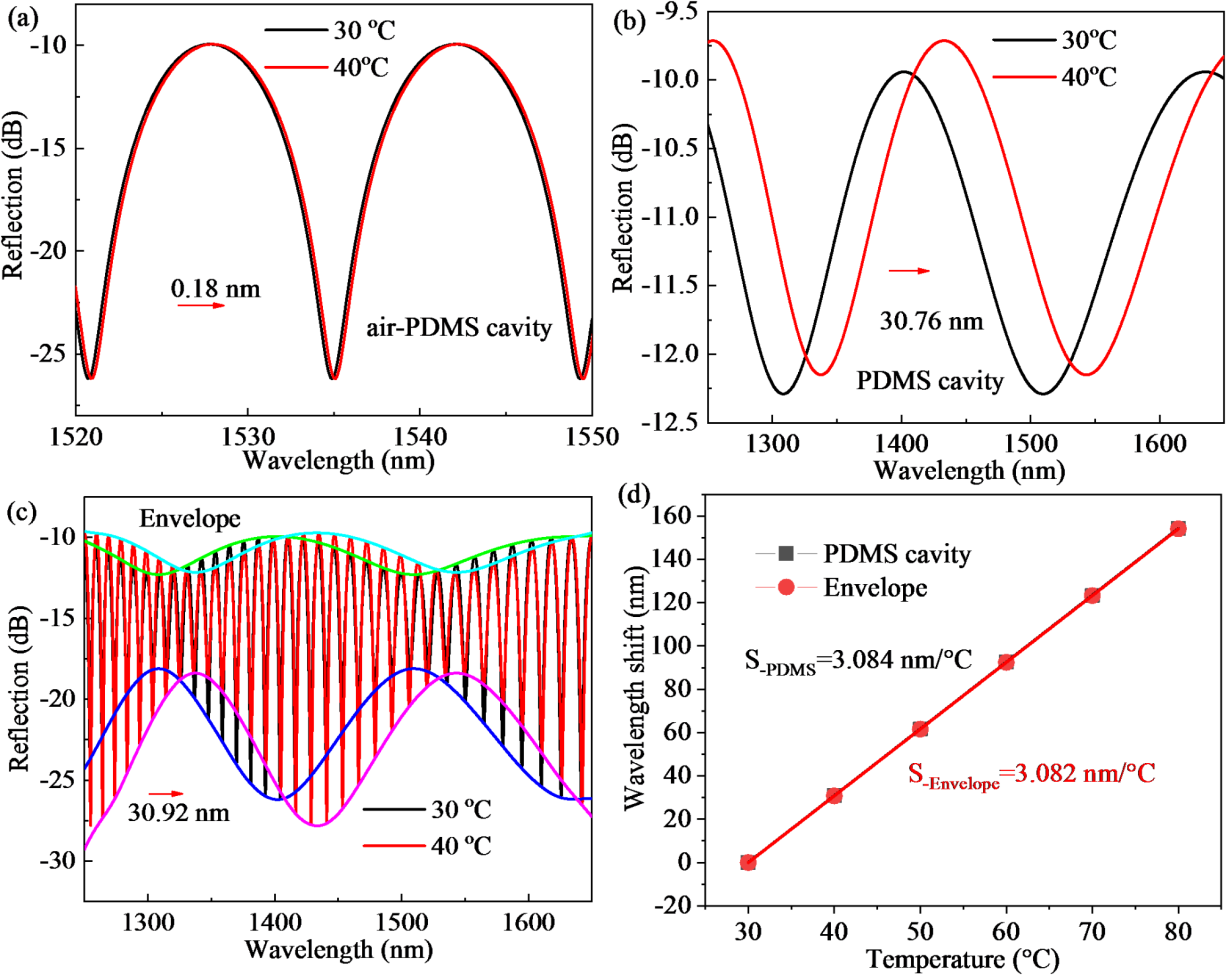
The simulated results of temperature from 30 ℃ to 40 ℃:(**a**–**c**) Wavelength shifts of air-PDMS cavity, PDMS cavity and envelope spectra; (**d**) The temperature sensitivities of PDMS cavity and envelope spectra.

According to the sensitivity calculation Eq. (3)^29^, it can be inferred that the temperature sensitivity expressions of a single PDMS polymer microcavity and mixed cavity of air and PDMS are Eqs. (4) and (5), respectively. By comparing Eqs. (4) and (5), it can be seen that the temperature sensitivity of air and PDMS mixing cavity is smaller than that of a single PDMS polymer microcavity because the length of the air and PDMS mixing cavity is greater than that of a single PDMS polymer microcavity. The temperature sensitivity of the Vernier envelope formed by the cascaded FPI optical fiber sensor is the multiplication of the air-PDMS mixed cavity’s sensitivity and the magnification (seeing Eq. (6)). By comparing Eqs. (4) and (6), it can be seen that the temperature sensitivities of a single PDMS polymer microcavity and the temperature sensitivity of the envelope spectrum are the same. Therefore, although the temperature sensitivity of the air-PDMS mixed cavity is amplified by the vernier effect. The value is equal to the temperature sensitivity of a single PDMS polymer microcavity, which is consistent with theoretical calculation. Then, we further explored the relationship between the FSR of a single PDMS polymer microcavity and the FSR of the Vernier envelope spectrum. The FSR of the Vernier envelope spectrum of the cascaded FPI with thin film is obtained by bringing the relevant parameter values into Eq. (7) (seeing Eq. (8)). By comparing Eq. (2) and Eq. (8), it can be seen that the FSR values of a single PDMS polymer microcavity and the Vernier envelope spectrum are the same. In order to verify above conclusion, the sensing performance of sample D is simulated according to actual preparation including the following parameter settings. The length of air cavity and PDMS cavity are 82.5, 3.7 μm with RI of 1, 1.4, respectively. As shown in Fig. 4, the temperature sensitivity of sample D has been theoretically simulated. The simulated spectra of air-PDMS cavity, PDMS cavity and envelope with wavelength shift of 0.18, 30.76 and 30.92 nm are shown in Fig. 4a–c, respectively when temperature varies from 30 ℃ to 40 ℃. From Fig. 4d, the wavelength shifts of PDMS cavity and envelope and temperature sensitivity are almost the same as temperature change.

4 $$S = \frac{{\partial \lambda }}{{\partial P}} = \lambda (\frac{1}{L}\frac{{\partial L}}{{\partial P}} + \frac{1}{n}\frac{{\partial n}}{{\partial P}})$$

5 $$S_{{PDMS(T)}} = \frac{{\partial \lambda }}{{\partial T}} = \lambda (\frac{1}{{L_{1} }}\frac{{\partial L_{1} }}{{\partial T}} + \frac{1}{{n_{1} }}\frac{{\partial n_{1} }}{{\partial T}}) = \frac{\lambda }{{n_{1} L_{1} }}\frac{{n_{1} \partial L_{1} + L_{1} \partial n_{1} }}{{\partial T}}$$

6 $$S_{{air - PDMS(T)}} = \frac{\lambda }{{n_{1} L_{1} + n_{2} L_{2} }}\frac{{n_{1} \partial L_{1} + L_{1} \partial n_{1} }}{{\partial T}}$$

7 $$S_{{env - TVE(T)}} = S_{{air - PDMS(T)}} M = \frac{\lambda }{{n_{1} L_{1} + n_{2} L_{2} }}\frac{{n_{1} \partial L_{1} + L_{1} \partial n_{1} }}{{\partial T}}(\frac{{n_{1} L_{1} + n_{2} L_{2} }}{{n_{1} L_{1} }}) = \frac{\lambda }{{n_{1} L_{1} }}\frac{{n_{1} \partial L_{1} + L_{1} \partial n_{1} }}{{\partial T}}$$

8 $$FSR_{{env - TVE}} = \frac{{FSR_{{hybrid}} FSR_{{air}} }}{{\left| {FSR_{{hybrid}} - FSR_{{air}} } \right|}} = \frac{{\lambda _{m} ^{2} }}{{2\left| {n_{1} L_{1} + n_{2} L_{2} - n_{2} L_{2} } \right|}}$$

9 $$FSR_{{env - TVE}} = \frac{{\lambda _{m} ^{2} }}{{2n_{1} L_{1} }}{\text{ }}$$

The change in cavity length of PDMS can be calculated using Eq. (9)^30^. From Eq. (9), we can know that PDMS length’s change (Δ*L*) is directly proportional to the Poisson’s ratio (*v*), radius of PDMS microcavity (*r*) and gas pressure change (Δ*G*), which is inversely proportional to the modulus of elasticity (*E*) and thickness of PDMS (*t*). When the external gas pressure increases, the PDMS polymer microcavity at the end of the sensor is elastically deformed by the force, resulting in a change in the cavity length of FPI_1_ and a negligible change in the refractive index of the medium. Equations (10) and (11) are formulas for calculating the gas pressure sensitivity of a single PDMS polymer microcavity and a mixed cavity of air and PDMS, respectively. The gas pressure sensitivity of the Vernier envelope formed by FPI_2_ and FPI_3_ is the product of the magnification and the gas pressure sensitivity of the air-PDMS. Equations (10),  (12) show that the gas pressure sensitivity of the Vernier envelope is equal to the gas pressure sensitivity of a single PDMS polymer microcavity.

10 $$\Delta L = \frac{{(1 - v)r^{2} }}{{2Et}}\Delta G$$

11 $$S_{{PDMS(G)}} = \frac{{\partial \lambda }}{{\partial G}} = \lambda (\frac{1}{{L_{1} }}\frac{{\partial L_{1} }}{{\partial G}}) = \frac{\lambda }{{n_{1} L_{1} }}\frac{{n_{1} \partial L_{1} }}{{\partial G}}$$

12 $$S_{{air - PDMS(G)}} = \frac{{\partial \lambda }}{{\partial G}} = \lambda (\frac{1}{{L_{1} + L_{2} }}\frac{{\partial L_{1} }}{{\partial G}}) = \frac{\lambda }{{n_{1} L_{1} + n_{2} L_{2} }}\frac{{n_{1} \partial L_{1} }}{{\partial G}}$$

13 $$S_{{env - TVE(G)}} = S_{{air - PDMS(G)}} M = \frac{\lambda }{{n_{1} L_{1} + n_{2} L_{2} }}\frac{{n_{1} \partial L_{1} }}{{\partial G}}(\frac{{n_{1} L_{1} + n_{2} L_{2} }}{{n_{1} L_{1} }}) = \frac{\lambda }{{n_{1} L_{1} }}\frac{{n_{1} \partial L_{1} }}{{\partial G}}$$

### Experimental verification of sensitivity

**Fig. 5:**
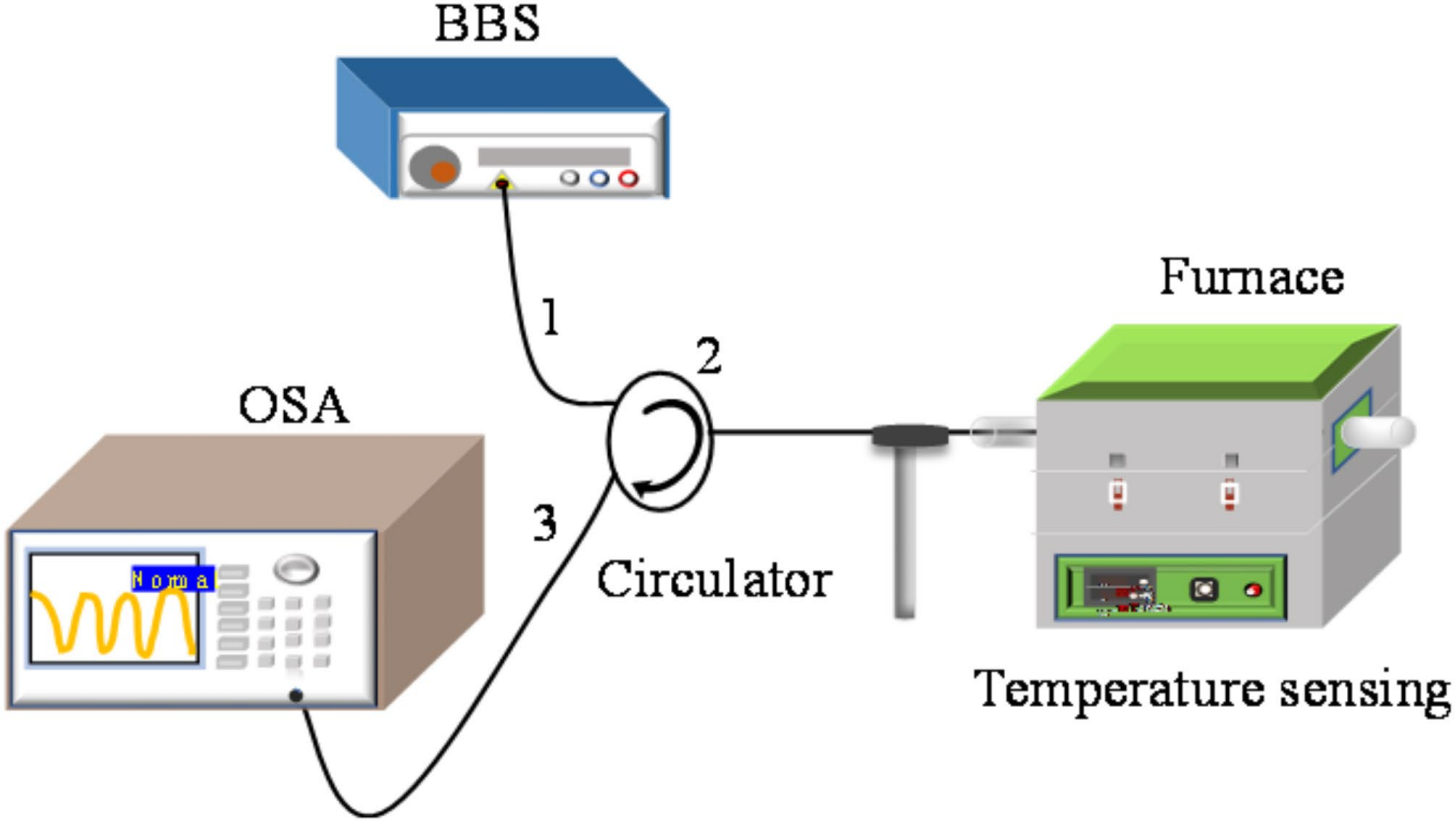
The experimental system for temperature test.

PDMS, as an excellent polymer material, has been widely applied in the field of temperature sensing and gas pressure measurement^31^. The length and RI of the PDMS cavity will change and make the length of air cavity also change as temperature varies, causing the wavelength shift or intensity variation of the envelope. The system for temperature measurement is presented in Fig. 5, which includes circulator, a broadband light source (BBS, Fiberlake Technology Co., Ltd, spectral range: 1250–1650 nm), the fabricated FPI, optical spectrum analyzer (OSA, AQ6370C, resolution: 0.02 nm), and high precision temperature control box (Labonce-6090VC). Then connect the three ports of the circulator with a BBS, and OSA, and the fabricated samples placed in a temperature control box, respectively. The reflective spectra at different temperature values are monitored and recorded by OSA. Temperature sensitivities of four sensors are experimentally tested to validate the theoretical reasoning.

**Fig. 6:**
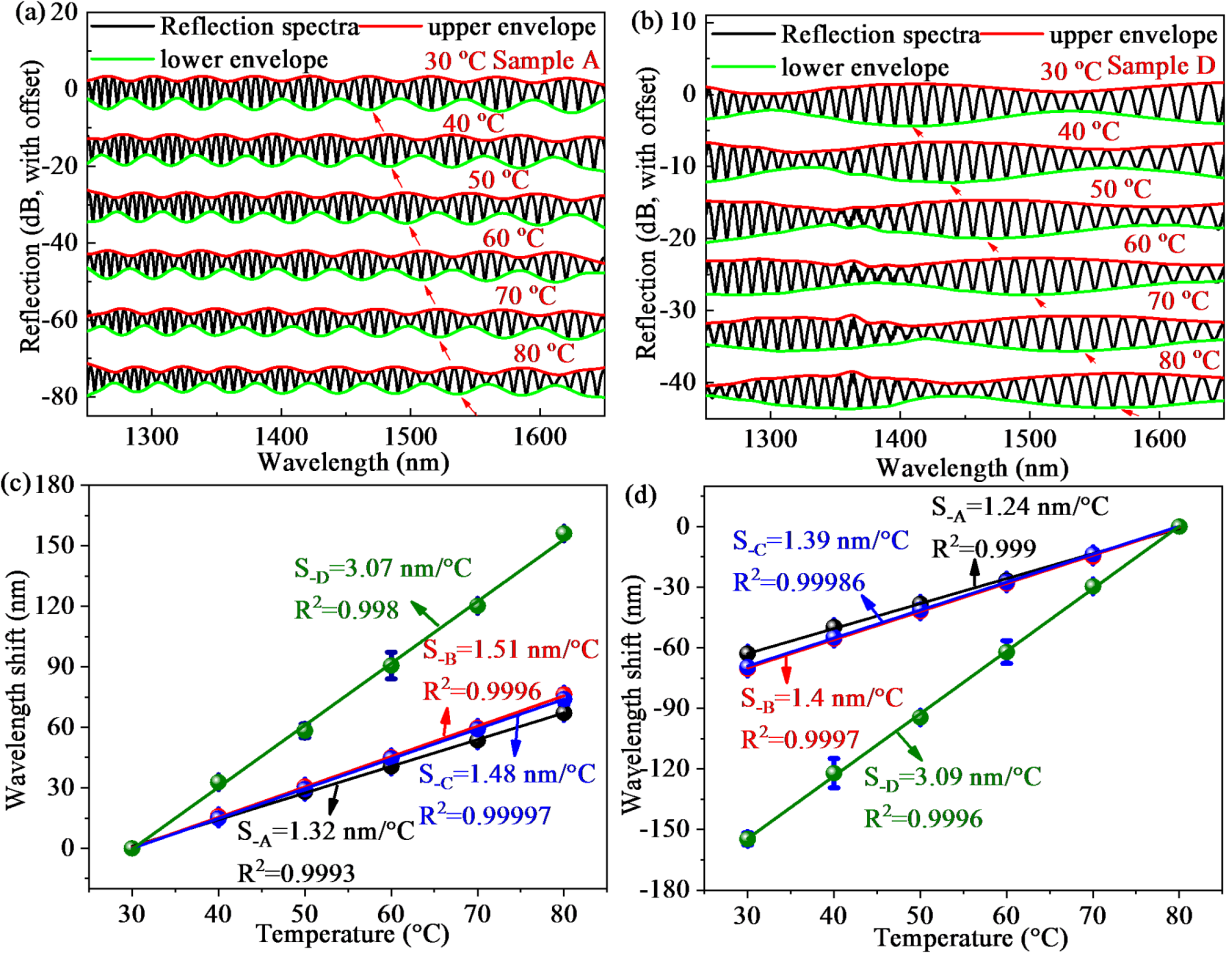
Reflection spectra at the temperature range of 30–80 ℃: (**a**) Sample A; (**b**) Sample D; The relationship between temperature and wavelength shifts for four samples: (**c**) Heating process; (**d**) Cooling process.

The reflective spectra of sample A and sample D are summarized in Fig. 6a,b when temperature rises from 30 ℃ to 80 ℃. As temperature increases, the envelope spectra monotonically shift towards the long wavelength direction. The reason for this phenomenon is that the increase in temperature triggers the increase in length and the decrease in RI of the PDMS cavity because of the thermo-optical effect and thermal expansion effect. The relationships between the wavelength shift and temperature during the heating process and cooling process are conducted in three experimental tests shown in Fig. 6c,d, which are both linearly variable. As presented in Fig. 6c, the samples A-D have average temperature sensitivities of 1.32, 1.51, 1.48, and 3.07 nm/℃ with small error bars and high linear correlation coefficients (LCCs) of 0.9993, 0.9996, 0.99997, and 0.998 when temperature varies from 30 ℃ to 80 ℃. Due to the similar parameters of the air cavity and air-PDMS mixed cavity from samples A-C, the temperature and gas pressure responses of the four samples are not significantly different. As temperature decreases from 80 to 30 ℃, the average temperature sensitivities are 1.24, 1.4, 1.39, and 3.09 nm/℃ with high linearity of 0.999, 0.9997, 0.99986, and 0.9996. Therefore, the average temperature sensitivity of the proposed mixed FPI during the heating process is almost the same as that during the cooling process, indicating the good repeatability and reversibility. In addition, the experimental data is consistent with theoretical calculation plotted in Fig. 4d, indicating the correctness of the result.

**Fig. 7:**
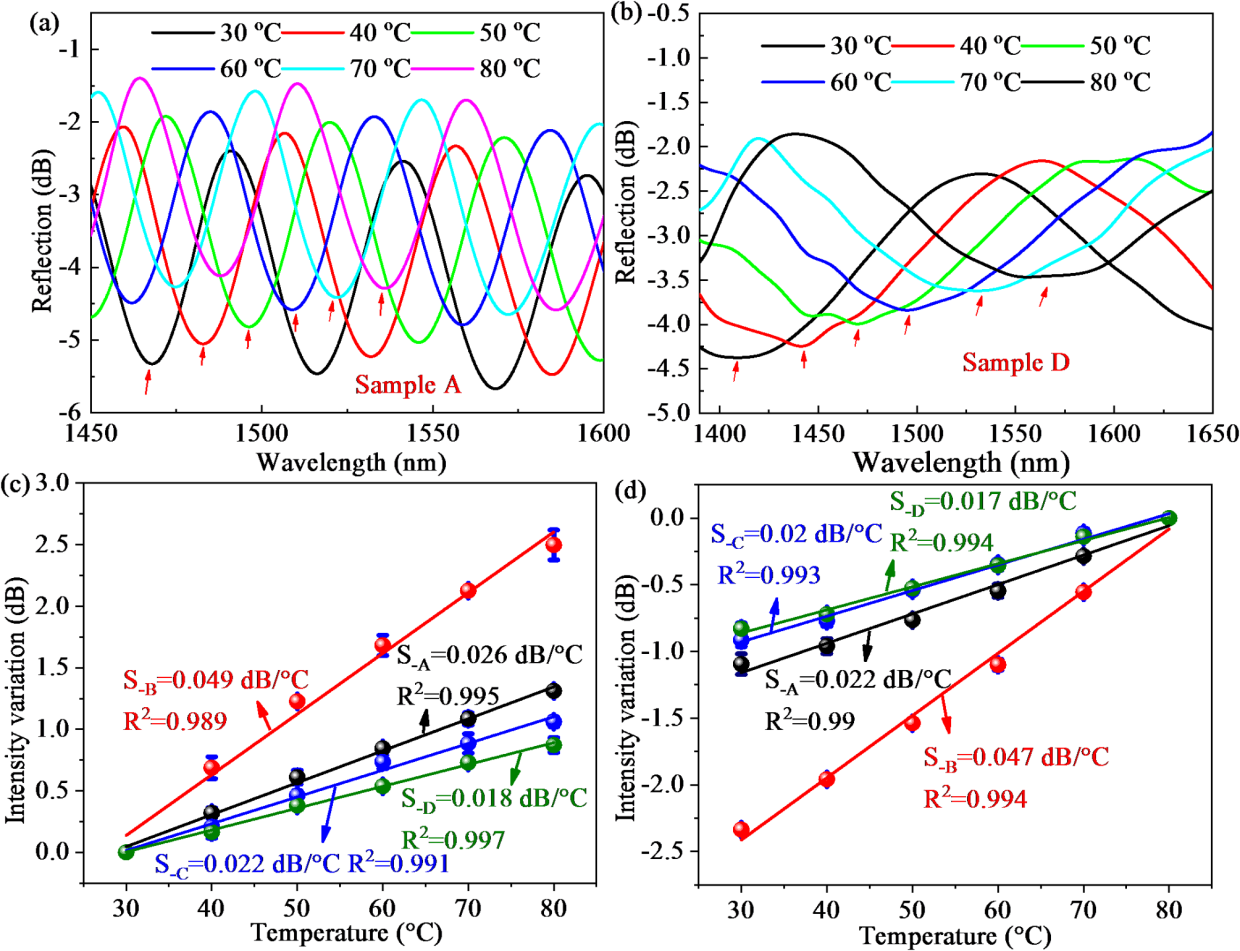
The intensity variation of the lower envelope at a temperature range of 30 –80 ℃: (**a**) Sample A; (**b**) Sample D; (**c**) The average temperature sensitivities from intensity variation during the heating process; (**d**) The average temperature sensitivities from intensity variation during the cooling process.

Not only wavelength shift but also intensity variation is investigated when temperature varies from 30 ℃ to 80 ℃. The relationships between temperature and intensity variations from the lower envelope of sample A and sample D is shown in Fig. 7a,b. The intensity of spectra varies monotonically with temperature change at range of 30 ℃ to 80 ℃. As presented in Fig. 7c,d, the samples A-D have average temperature sensitivities of 0.026, 0.049, 0.022, and 0.018 dB/℃ from intensity variation as temperature increases from 30 to 80 ℃, which agree well with the cooling process with sensitivities of 0.022, 0.047, 0.02, and 0.017 dB/℃, respectively.

**Fig. 8:**
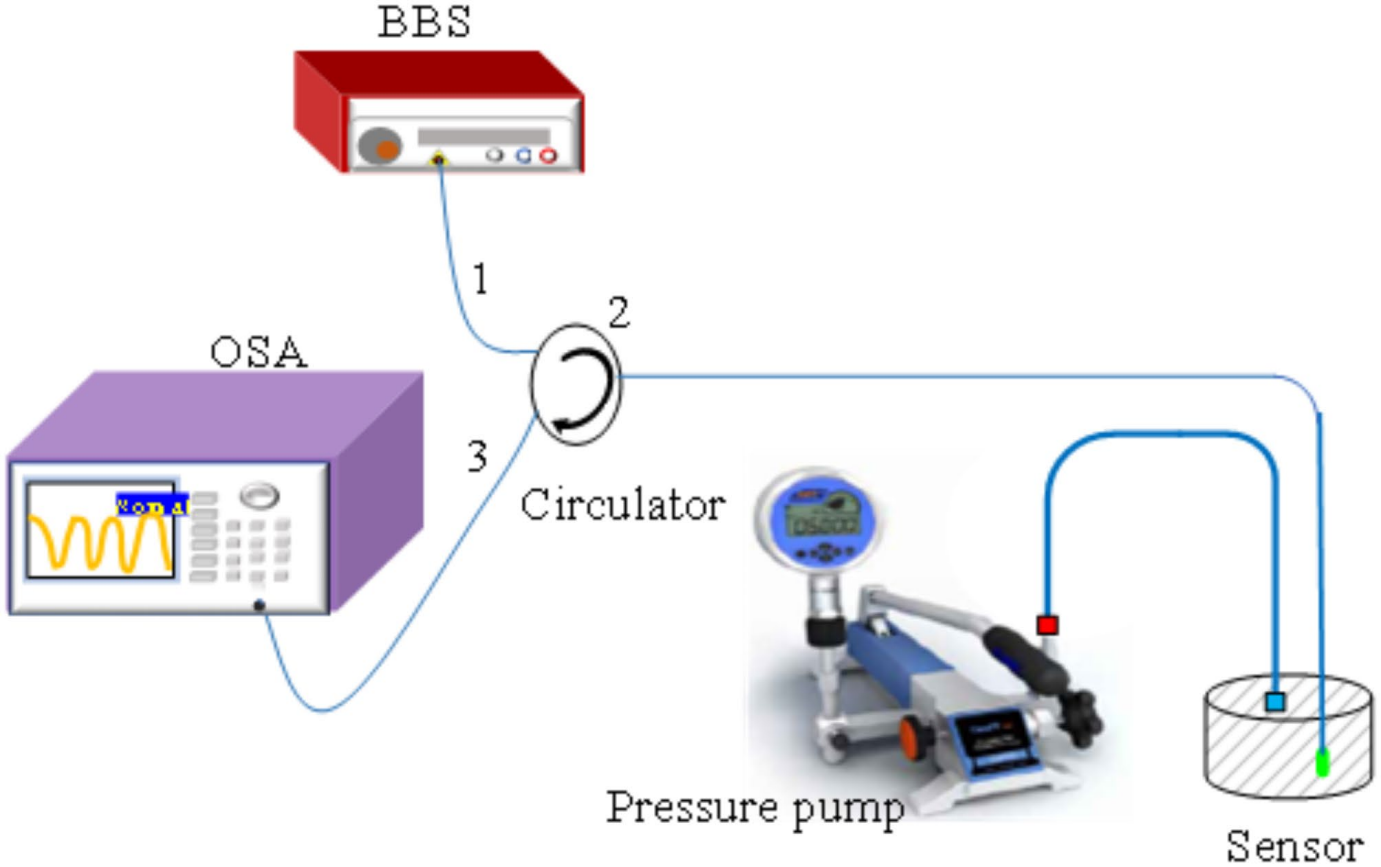
The experimental setup of gas pressure monitoring, including a BBS, OSA, circulator, pressure pump (ConST-162), gas chamber, and the designed sensing probe.

**Fig. 9:**
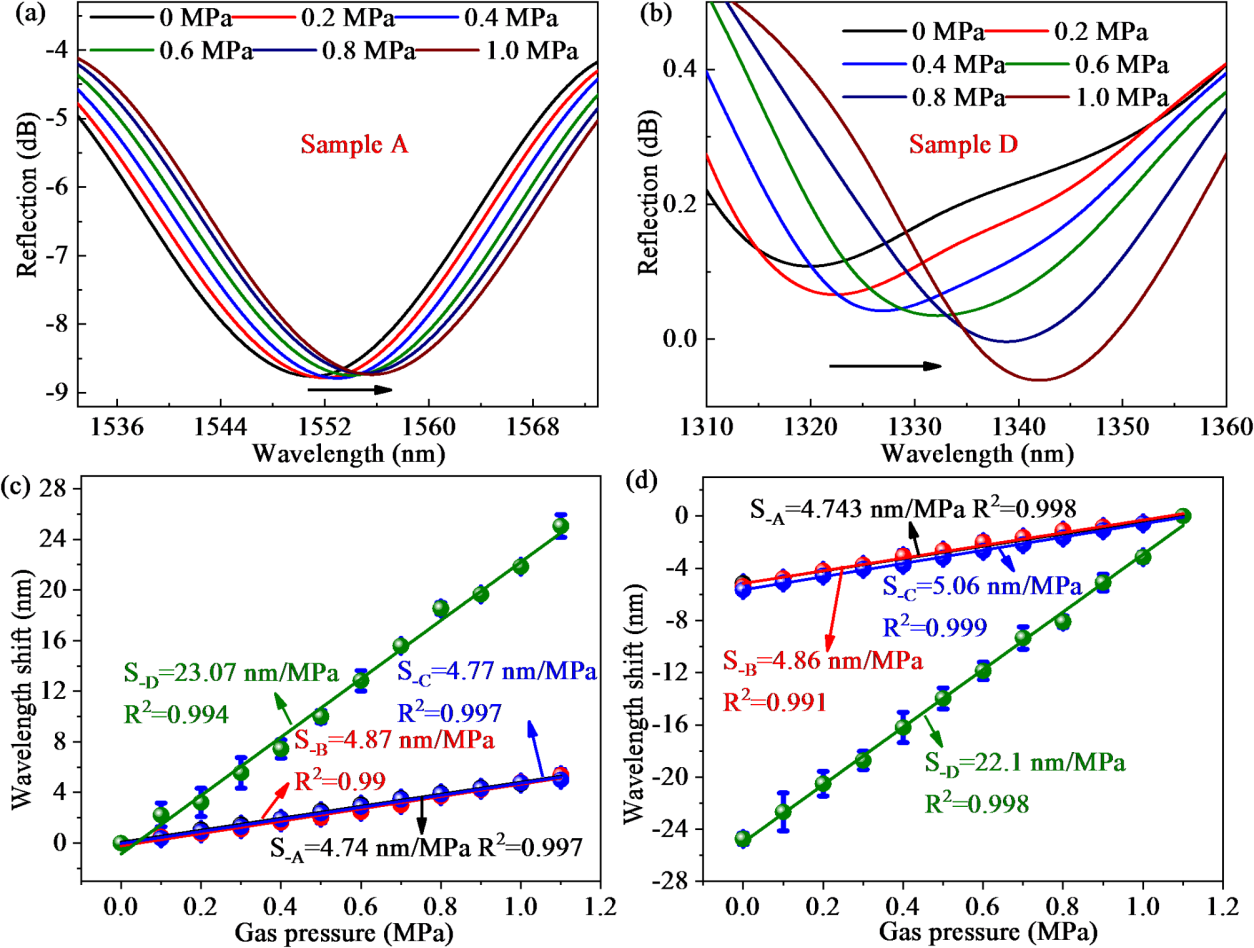
Spectral response of hybrid FPI at a range of 0-1.1 MPa: (**a**) Sample A; (**b**) Sample D; (**c**) Summarized gas pressure sensitivities of four samples when gas pressure increases from 0 to 1.1 MPa; (**d**) Summarized gas pressure sensitivity of four samples during the process of gas pressure drop.

The four samples are successively embedded in the self-made gas chamber, where gas pressure can be controlled by a pressure pump. The system for gas pressure sensing is illustrated in Fig. 8. As the gas pressure in the chamber increases, the PDMS microcavity undergoes slight deformation and changes the PDMS microcavity length, resulting in a spectral shift^32^. Once the wavelength shift is calibrated, the change in surrounding gas pressure will be determined. The spectral responses of the envelope from sample A and sample D are shown in Fig. 9a,b when gas pressure increases from 0 MPa to 1.1 MPa, which exhibits a “redshift” trend. Notably, the wavelength shift of sample D is much greater than that of sample A within the same range of gas pressure changes, indicating that the gas pressure sensitivity of sample D is higher than that of sample A. To ensure the accuracy of the gas pressure measurement results, three repeated tests were conducted to obtain the error bar. As presented in Fig. 9c, the gas pressure sensitivities of sample A-D are 4.74, 4.87, 4.77, and 23.07 nm/MPa with high linearity of 0.997, 0.99, 0.997, and 0.994, respectively during the process of increasing gas pressure. As presented in Fig. 9d, the gas pressure sensitivities of sample A-D are 4.743, 4.86, 5.06, and 23.07 nm/MPa with little error bars and high LCCs of 0.998, 0.991, 0.999, and 0.998, respectively when gas pressure decreases from 1.1 MPa to 0 MPa. Thus, the gas pressure sensitivity during the process of increasing gas pressure is consistent with the process of decreasing gas pressure, indicating the proposed sensor probe has excellent repeatability and reversibility to gas pressure measurement.

**Fig. 10:**
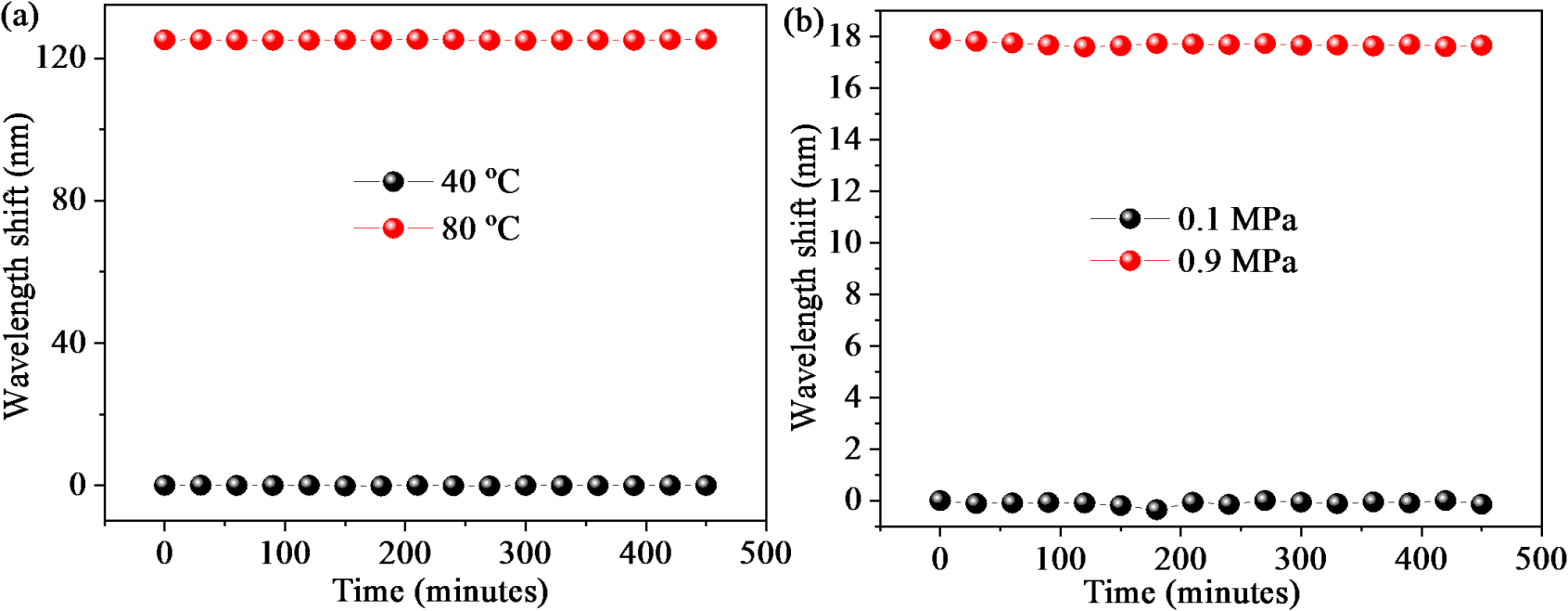
Stability test of sample D: (**a**) Monitoring wavelength deviations of the envelope at 40℃, 80℃ within 450 min; (**b**) Monitoring wavelength deviations of the envelope at 0.1 MPa, 0.9 MPa within 450 min.

As plotted in Fig. 10a, the temperature stability of sample D was experimentally investigated by monitoring the wavelength deviations of the envelope at 40 ℃, and 80 ℃ within 450 min. The maximum wavelength shift is only 0.16 nm (corresponding to 0.05 ℃), revealing that sample D has good temperature stability. In addition, the wavelength shifts of sample D are continuously tracked when gas pressure values are 0.1 and 0.9 MPa, respectively. As plotted in Fig. 10b, the maximum spectral shift of sample D at 0.1 and 0.9 MPa is 0.349 nm, which is equivalent to the gas pressure change of 0.015 MPa. Thus, we can conclude that gas pressure stability of the proposed mixed FPI is relatively good.

## Discussion

Generally, the measurement limit corresponds to three times of maximum wavelength variation from stability test^33^. Thus, the temperature detection limit of the proposed sensor is estimated as 0.16 ℃ using Eq. (1). The value of 0.046 MPa is obtained according to the definition of the gas pressure measurement limit expressed as Eq. (2).

14 $$LOD_{T} = \frac{{3\sigma }}{S} = \frac{{0.48\,\,nm}}{{3.07\,\,nm/(^\circ C)}} = 0.16\,\,^\circ C$$

15 $$LOD_{P} = \frac{{3\sigma }}{S} = \frac{{1.05\,\,nm}}{{23.07\,\,nm/(MPa)}} = 0.046\,\,MPa$$

Table 2 compares the sensing performance of several different sensor structures from the following five aspects: the sensor types, materials, temperature sensitivity (T. s.), gas pressure sensitivity (G. p. s.), and with or without Vernier effect. From table 2, the designed FPI presented in this work has much higher temperature and gas pressure sensitivities than other sensor structures because of the ultra-thin PDMS film thickness and sensitivity amplification effect by employing the Vernier effect. In addition, the FPI proposed in this paper is compact, low-cost, and easy to prepare.

**Table 2:** Temperature and gas pressure sensing performance of different optical fiber sensors.

Sensor structures	Polymermaterials	T. s. (nm/℃)	G. *p*. s. (nm/MPa)	Vernier effect	Reference (Year)
Dual-cavity FPI	PDMS	2.62	20.63	No	^34^ (2020)
Diaphragm-Free FPI	No	0.0148	4.28	No	^35^ (2018)
Polymer-capped FPI	UV glue	0.249	1.13	No	^19^ (2015)
Tapered seven-core fiber and PDMS cap	PDMS	-0.22	2.27	No	^36^ (2023)
Polymer optical fiber grating	No	0.0166	0.48	No	^37^ (2021)
Fabry-Perot silica-microprobe	No	0.0043	16.3536	No	^38^ (2022)
FPI with core offset fusion of HCF	No	0.0051	4.314	No	^39^ (2019)
Dual FPI based on suspended core fiber	No	0.0117	3.8	No	^40^ (2021)
MZI	No	0.046	8.239	No	^41^ (2015)
FPI-based SMF cascade FBG	No	0.2	13.1	No	^42^ (2023)
Side-polished fiber microcavity	PDMS	−1.19	−3.96	No	^43^ (2022)
Hybrid FPI	PDMS	3.07	23.07	Yes	This work

## Conclusion

In summary, a novel FPI structure with thin film based on the Vernier effect formed by cascading the SMF, HCF, and PDMS microcavity is proposed, and experimentally demonstrated. The OPL of the PDMS microcavity is much smaller than that of the air cavity due to the fact that the length of the PDMS microcavity is much smaller than the length of the air cavity. Therefore, the Vernier effect was obtained via engineering the OPLs between the air cavity and the air-PDMS mixed cavity. In addition, temperature and gas pressure measurements have been achieved by utilizing the material properties of the PDMS. By choosing the ultra-thin PDMS film with a thickness of 3.7 μm and 82.5 μm HCF, the sensor has high temperature and gas pressure responses with value of 3.07 nm/℃ and 23.07 nm/MPa and corresponding *M* is 17. The sensitivity of the cascaded FPI with thin film based on Vernier effect depends on the sensitivity of the individual thin film cavities. Furthermore, the proposed sensors feature the virtues of simple fabrication, compact structure, strong robustness, and high sensitivity.

## Methods

### Fabrication of FPI sensor

**Fig. 11:**
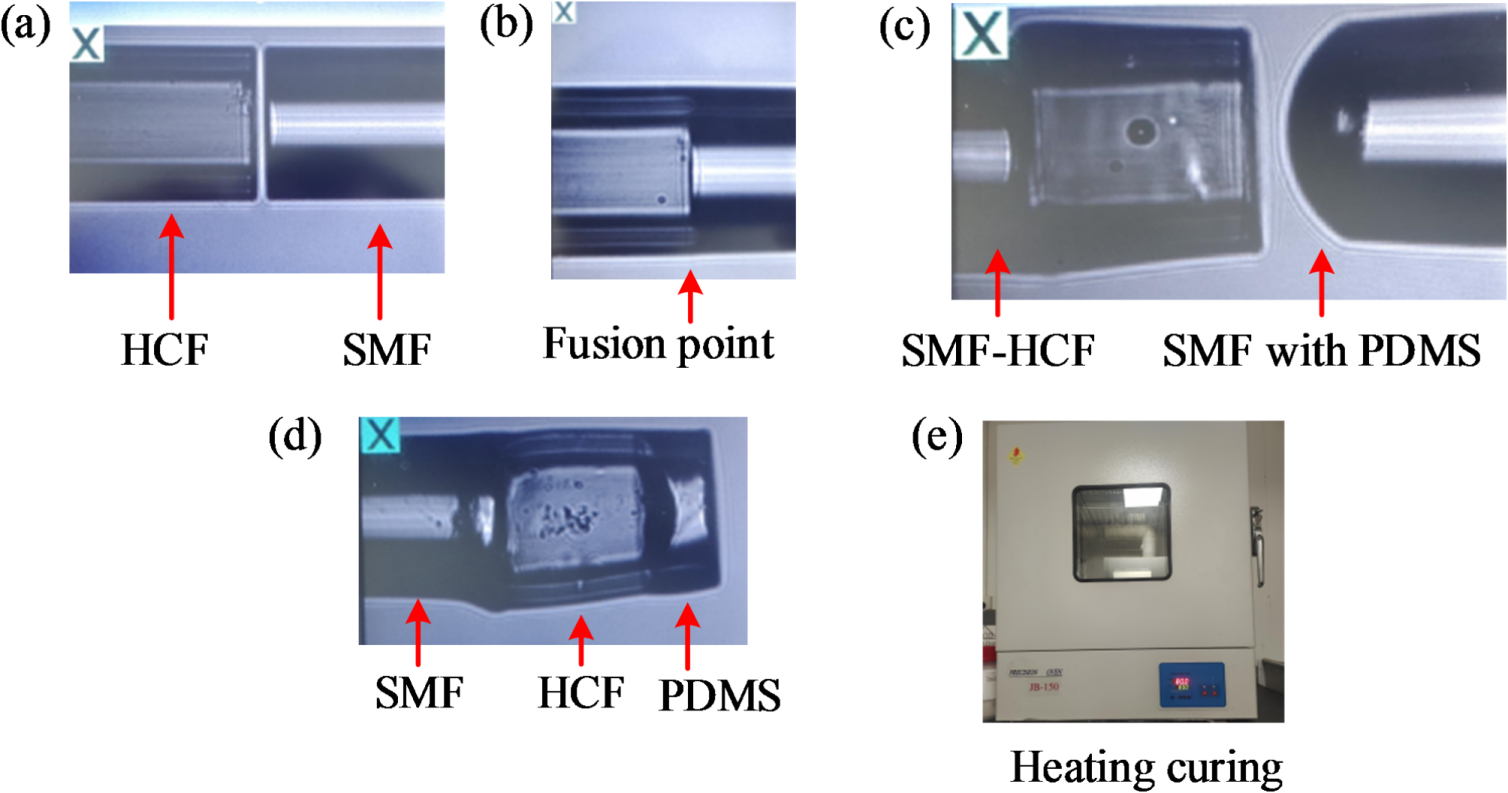
Detailed preparation process of sensor probe.

The FPI sensor fabrication process is shown in Fig. 11. Firstly, the HCF is cleaved and clamped on the left holder of the fusion splicer (Fujikura, 80 S+). A cleaved single-mode fiber (SMF) with a flat end face is fixed by the clamp on the right side of the splicer. The image of this operation process is shown in Fig. 11a. Then the HCF is spliced with SMF using manual mode (the discharge power: -80 bit, discharge time: 400 ms), and the spliced fiber is shown in Fig. 11b. Secondly, the other side of the HCF is cleaved with flat end. To control length of the cleaved cavity, the whole cleaving process is conducted under an optical microscope with high precision 3D displacement platform. Another cleaved SMF was immersed into a mixed liquid (PDMS) consisting of Sylgard 184 silicone elastomer base and a curing agent with a ratio of 5:1. Some PDMS liquid droplet was adhered on the SMF end face due to the surface tension forming a cap shape, which is champed by the right holder of the fusion splicer. Figure 11c shows the image of the PDMS cap aligned with SMF-HCF structure. The PDMS cap is then successfully transferred to the HCF by controlling the motor of the splicer as shown in Fig. 11d. The thickness of the PDMS film could be controlled precisely by repeating the above process and calibrating with the help of the microscope. Finally, the prepared sensor structure was vertically placed in a thermostatic vacuum drying oven at 80 ℃ for five days to form a PDMS microcavity (Fig. 11e). A more uniform and smooth solid surface and a greater sensor contrast can be obtained by vertical placement of FPI.

**Fig. 12:**
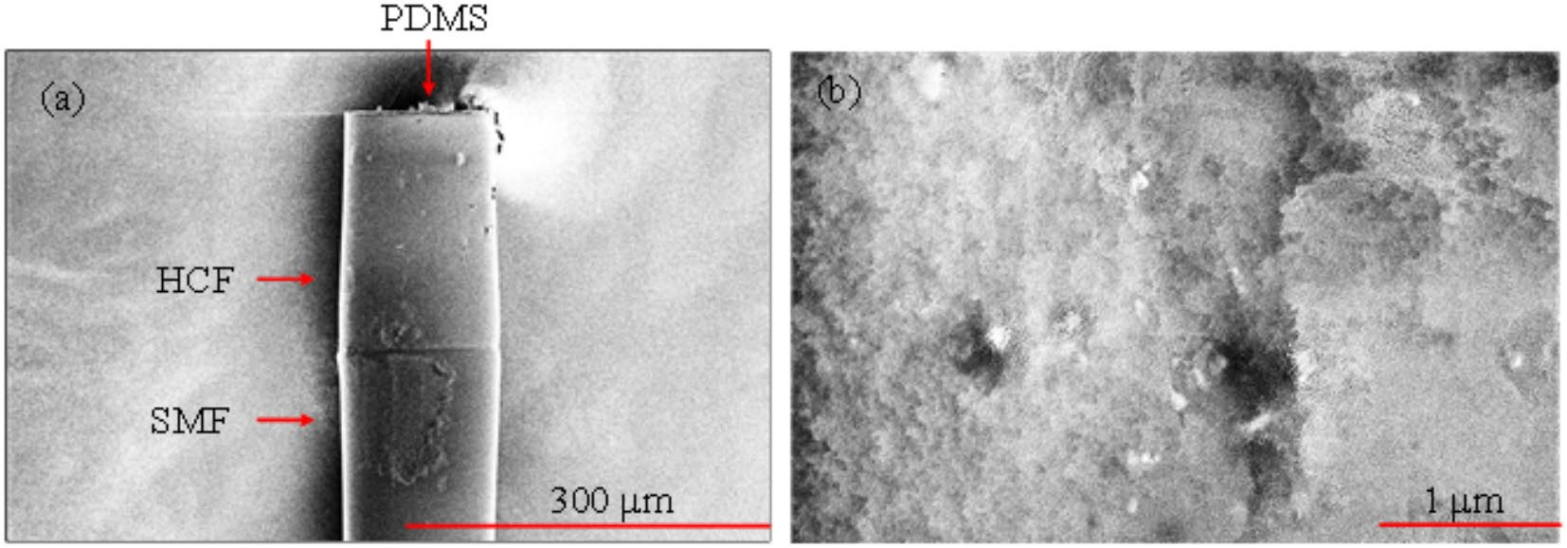
SEM images: (**a**) The fabricated sensor probe; (**b**) Morphology and topographical details of the PDMS microcavity.

Scanning electron microscopy (SEM, Thermo Scientific Apreo) images of the fabricated sensor probe is presented in Fig. 12a, indicating SEM can only observe the surface of the sensor probe. The morphology and topographical details of the PDMS microcavity is presented in Fig. 12b.

## Data Availability

The datasets used and/or analysed during the current study available from the corresponding author on reasonable request.
